# Internet-of-Things Skills Among the General Population: Task-Based Performance Test Using Activity Trackers

**DOI:** 10.2196/22532

**Published:** 2020-11-18

**Authors:** Pia S de Boer, Alexander J A M van Deursen, Thomas J L van Rompay

**Affiliations:** 1 Department of Communication Science University of Twente Enschede Netherlands

**Keywords:** internet of things, activity tracker, mobile phone, skills, digital divide, performance test

## Abstract

**Background:**

The health internet-of-things (IoT) can potentially provide insights into the present health condition, potential pitfalls, and support of a healthier lifestyle. However, to enjoy these benefits, people need skills to use the IoT. These *IoT skills* are expected to differ across the general population, thereby causing a new digital divide.

**Objective:**

This study aims to assess whether a sample of the general Dutch population can use health IoT by focusing on data and strategic IoT skills. Furthermore, we determine the role of gender, age, and education, and *traditional* internet skills.

**Methods:**

From April 1, 2019, to December 12, 2019, 100 individuals participated in this study. Participants were recruited via digital flyers and door-to-door canvassing. A selective quota sample was divided into equal subsamples of gender, age, and education. Additional inclusion criteria were smartphone possession and no previous experience of using activity trackers. This study was conducted in 3 waves over a period of 2 weeks. In wave 1, a questionnaire was administered to measure the operational, mobile, and information internet skills of the participants, and the participants were introduced to the activity tracker. After 1 week of getting acquainted with the activity tracker, a task-based performance test was conducted in wave 2 to measure the levels of data IoT skills and the strategic IoT skill component—*action plan construction*. A week after the participants were asked to use the activity tracker more deliberately, a performance test was then conducted in wave 3 to measure the level of the strategic IoT skill component—*action plan execution*.

**Results:**

The participants successfully completed 54% (13.5/25) of the data IoT skill tasks. Regarding strategic IoT tasks, the completion rates were 56% (10.1/18) for action plan construction and 43% (3.9/9) for action plan execution. None of the participants were able to complete all the data IoT skill tasks, and none of the participants were able to complete all the strategic IoT skill tasks regarding action plan construction or its execution. Age and education were important determinants of the IoT skill levels of the participants, except for the ability to execute an action plan strategically. Furthermore, the level of information internet skills of the participants contributed to their level of data IoT skills.

**Conclusions:**

This study found that data and strategic IoT skills of Dutch citizens are underdeveloped with regard to health purposes. In particular, those who could benefit the most from health IoT were those who had the most trouble using it, that is, the older and lower-educated individuals.

## Introduction

### Background

The internet has undergone numerous changes over the years. It went from a medium restricted to reading content (web 1.0) to a web-based environment where users can create, store, and share content themselves (web 2.0). These functions were further developed by introducing semantics to smoothen the interaction with the internet (web 3.0). In the most recent iteration (web 4.0), objects are added to the network of interconnected people [1]. This development, known as the internet-of-things (IoT), uses the internet to form a network of ubiquitous everyday objects that can sense and analyze their environments, communicate this information to both people and other objects, and use this information to make autonomous decisions [2]. Some of the most common consumer IoT appliances can be found in the health domain [3]. Applications of the IoT include, for example, telemedicine and wearables collecting physiological data. Health IoT has the potential to provide insights into the present health condition, potential pitfalls, and support a healthier lifestyle [2]. Additional (future) benefits include lifestyle management when undergoing treatment, support for health-related decisions, and cost savings on matters such as health insurance [4]. However, to benefit from health IoT appliances, people must be able to cope with the continuous data stream, make decisions based on these data, and evaluate and act upon data-driven decisions made by the IoT [2].

In 2018, 31% of the Dutch population used at least one health IoT device [5]. Almost half of these users were highly educated and aged between 18 and 35 years (44% and 45%, respectively). The most commonly used IoT device was the activity tracker (11%), a wearable device that continuously gathers data on physical activities (eg, number of steps taken, distance covered, and stairs climbed), intensity of activities (eg, by heart rate and calories), and recovery from intense activities (eg, through sleep). Users have to make sense of the collected data, that is, recognize what they were doing when certain data were collected, understand how data are presented, extract meaning from the data, and assess the reliability of the data (eg, was a heart rate peak because of intense exercise or a device malfunction?). In addition to understanding the data, users must know how to make informed decisions based on these data and act accordingly. For instance, to improve stamina, users can decide to use the activity tracker to adapt their training to their heart rate instead of sticking to a predetermined training scheme. In sum, the activity tracker allows users to train more effectively but requires advanced skills to fully capitalize on its potential benefits [2].

In line with this observation, previous research in the realm of the digital divide has indicated that internet skills are a primary determinant of eHealth use and outcomes [6-8]. As these internet skills proved to be relevant throughout web developments, including that of the IoT [9], we expect to see similar patterns of skill inequality regarding the use of health IoT. Accordingly, the older population, people from a lower socioeconomic status, and those with disabilities or health issues are expected to have lower levels of skills necessary to operate the IoT [2]. This suggests that the people who could potentially benefit most from the health IoT are least likely to get the most out of these IoT devices. In this study, we aim to answer the following questions: (1) what are the levels of IoT skills of Dutch citizens? and (2) what personal characteristics (gender, age, and level of educational attainment) and what internet skills determine the levels of IoT skills of individuals? The questions are addressed by a 3-wave study wherein the participants—initially nonusers of health IoT—are provided with an activity tracker for 2 consecutive weeks.

### IoT Skills

From previous research on digital inequality, we know that internet skills are a key factor for beneficial internet use [6-8,10-13]. At first, this need for skills seems less relevant for using the IoT, as a primary characteristic of the IoT system is that it operates rather autonomously. However, Van Deursen and Mossberger [2] argue that skills remain relevant for beneficial IoT use, as users must be able to cope with the ambiguity of the IoT system, the vast amount of data, the decisions made for them, and the increased privacy and security risks IoT use brings. The complexity of the IoT system questions the possibility of a fair distribution of costs and benefits and equal opportunities for benefiting from the IoT [14]. To study skill inequality regarding the IoT, the skills of previous web developments serve as a starting point [2,9].

The skills necessary to use the internet can be divided into operational, information, communication, creative, and strategic internet skills [8]. In a previous survey research, internet skills were found to contribute to IoT skills (when considered as a unidimensional construct; [9]). The distinction between different internet skills, however, can also be applied to IoT skills. In this study, we focus on 2 types of IoT skills that are apparent through the entire process of using the IoT: data IoT skills and strategic IoT skills (corresponding to information and strategic internet skills, respectively).

#### Data IoT Skills

Data IoT skills are required to make sense of the data that are continuously gathered by the IoT without user interference. As IoT devices gather data autonomously, data IoT skills focus on deducting where the data are coming from, interpreting the data, and assessing their reliability and relevance to the context in which they are used [15,16]. By introducing objects to the network, we have moved away from the ability to use a search engine and are headed toward internet skills wherein data literacy—“the component of information literacy that enables individuals to access, interpret, critically assess, manage, handle and ethically use data” [17]—becomes more important. The main difference between information internet skills and data IoT skills lies in the greater complexity of handling (big) data compared with other information types [18]. Users must be able to find specific data in a continuously increasing data set, combine and present the data in a clear overview (eg, graph or summary), and connect the data to events in the (offline) environment. Textbox 1 provides a more detailed overview of data IoT skills (based on internet skills described by Iordache et al [19] and 21st century digital skills described by Van Laar et al [20]).

**Data and strategic internet-of-things skills definitions.**

Data internet-of-things (IoT) skills is the ability to:

- determine when data are needed [17]
- set out a plan for how data are gathered [19,20]
- recognize the available data sources [17,19]
- critically assess data and their sources [17,19,20]
- select relevant data [17,20,21]
- present quantitative data [17]
- extract meaning from data [17,19,20]
- identify the context in which data are produced and used of reused [17,22]

Strategic IoT skills is the ability to:

- set a realistic goal [20,23]
- recognize how IoT can help to reach the goal [20,23]
- combine data with previous measurements, prior knowledge, and other information sources to draw conclusions [17,19,20,24]
- evaluate proposed actions and autonomous decisions of IoT devices [19-21]
- make data-based decisions regarding the goal [19-21,23]
- reflect on progress made toward the goal [23]

#### Strategic IoT Skills

Strategic IoT skills are necessary to use the data to benefit from the IoT system. Strategic IoT skills broadly follow the 4 steps of decision making that are considered in studies about internet skills: goal orientation, taking required actions, making decisions, and implementing those decisions and gaining benefits from those decisions [25]. As such, strategic IoT skills enable users to recognize how the IoT can help them reach a personal goal; combine data, previous knowledge, and other information sources to make informed decisions toward the goal; implement these decisions by performing goal-oriented actions; and reflect on the progress made toward the goal. In addition to the implementation and reflection on their own decisions, IoT users must be able to evaluate actions proposed or autonomously undertaken by the IoT and act upon these propositions. Textbox 1 provides an overview of the strategic IoT skills.

### IoT Skill Determinants

Skills enable users to understand, interpret, and act upon the data and actions generated by the IoT. However, the possession of these skills is likely to differ among people. Research on internet skills has, for instance, long shown that education is an important resource for internet skills and that the older population has more problems using the internet [26,27]. These differences in skill levels are expected to be even more pronounced for the IoT because of its complexity and potential impact [28]. This is troublesome because people who rely the most on health-related services are likely to possess the lowest skill levels to use the health IoT for health support [2]. In addition, the big data generated by the IoT reinforce existing biases, as not every group (eg, racial and ethnic groups, disabled individuals, older individuals, and poor individuals) is represented, that is, present data only include people using the IoT and using it correctly [29].

For the internet, many determinants have been found to influence skills [30]. Most commonly studied determinants are sociodemographic and socioeconomic determinants, followed by motivational determinants. As this study is the first inventory of IoT skills, we start with the roles of gender, age, and education to answer the second research question, that is, what personal characteristics determine the levels of IoT skills of individuals? Furthermore, we study the role played by the levels of internet skills in possessing IoT skills.

Regarding gender and internet skills, the findings are inconsistent. Most self-evaluations in surveys found that men possess more internet skills than women, which has often been linked to earlier adoption and more extensive use of the internet [13,31-33]. However, other research found that men and women do not differ in their abilities but that women tend to underestimate their skills when compared with men [34,35]. In line with this argument, no differences were found between men and women in actual performance tests [7,26]. Furthermore, as education plays an important role when considering internet skills, a lack of gender differences can be expected, as in the Netherlands, gender differences within education have, to a large extent, disappeared [36]. Therefore, the following was hypothesized for IoT skill levels:

- *H1: There are no differences in data and strategic IoT skill levels between men and women.*

In general, older individuals experience more problems in using the internet, as they did not have the opportunity to acquaint themselves with the internet at an early age [37], have less access to support [8], and are hindered by mental and physical conditions [38]. These lower skill levels are also expected when using the health IoT, as usability research has shown that older individuals do not operate activity trackers beyond basic functions and that they have difficulty integrating the wearable device in their exercise planning, including goal setting [39]. Therefore, we hypothesize the following:

- *H2: Age contributes negatively to data and strategic IoT skills.*

Regarding educational attainment, those with higher levels possess more advanced internet skills [8,30] and are better able to keep up with technological advancements, resulting in greater inequality between themselves and lower-educated individuals who are unable to keep up [40]. We expect these differences in an IoT environment to become even larger, as a complex system requires even more cognitive capabilities [30,41]. We hypothesize the following:

- *H3: Education contributes positively to data and strategic IoT skills.*

Internet skills are expected to remain relevant for the possession of IoT skills, the same way as traditional literacy (eg, reading, writing, and understanding texts) has remained important for internet skills [9]. Operational and mobile internet skills are still needed for operating the IoT platform (website or app) and changing settings. In addition, information internet skills remain relevant as finding and selecting the correct web page and interpreting information remain relevant skills for selecting and interpreting the correct data in the IoT system. Furthermore, using the IoT is a matter of interpreting the data to act strategically. Therefore, operational, mobile, and information internet skills are hypothesized to be predictors of IoT skills:

- *H4: (a) Operational, (b) mobile, and (c) information internet skills contribute positively to data and strategic IoT skills.*

## Methods

### Recruitment

In this study, participants were recruited via the distribution of a (digital) flyer on social media and by door-to-door canvassing. Via the flyer, individuals were referred to a website created for the purpose of this study. The website contained more information about the study and participation in the study. It also included an option to sign up for the study. After signing up, potential participants were selected (those who signed up first had priority) based on quota sampling for gender, age, and educational attainment (low-middle-high). Additional inclusion criteria were that the participants were in possession of a smartphone and had no previous experience of using activity trackers. Of the 314 signups, 100 individuals were invited via phone to participate, and appointments were planned. These participants received a confirmation email with the appointments and their home address as the agreed research location. The participants were promised Eur €50 (US $60) for their participation in 3 research sessions of approximately 1.5 hours each.

Table 1 contains the number of participants and their distribution in terms of gender, age, and education.

**Table 1:** Distribution of the participants by gender, age, and education.

Characteristics		Value, n (%)
**Gender**		
	Male	48 (48.0)
	Female	52 (52.0)
**Age (years)**		
	18-29	24 (24.0)
	30-39	26 (26.0)
	40-54	24 (24.0)
	55-80	26 (26.0)
**Education**		
	Low	33 (33.0)
	Middle	34 (34.0)
	High	33 (33.0)

### Measures and Procedure

To answer the research questions, a 3-wave study was (physically) conducted wherein the participants—initially nonusers of health IoT—were provided with an activity tracker (Fitbit Charge 3; Fitbit Inc) for 2 consecutive weeks. In wave 1, the participants were introduced to the activity tracker for the first time. Hence, all the participants started out with no previous experience in using activity trackers. After 1 week of getting acquainted with the activity tracker, a task-based performance test was conducted in wave 2 to measure IoT skills. A week after the participants were asked to use the activity tracker more deliberately, a second performance test was then conducted in wave 3. Performance testing yields a direct measure of IoT skills. Although highly labor intensive, these tests are most valid and provide a realistic view of people’s actual IoT skills [25,42,43]. The activity tracker collected data on exercising (eg, steps, floors, distance, active minutes, calories, training, and heart rate) and sleeping habits (eg, sleep duration, sleep phases, and sleep schedule) of the participants. The participants had access to the data in the corresponding Fitbit app.

The study was conducted from April 1, 2019, to December 12, 2019, and took place at the homes of the participants. Before the first wave, a 5-min questionnaire was administered on the web to gather personal information. The participants were asked for their birth year, gender, level of educational attainment, and experience using an activity tracker.

#### Wave 1

The first wave started with a printed offline questionnaire to measure the levels of operational, mobile, and information internet skills of the participants using the corresponding items of the Internet Skills Scale [44]. To respond to the items, a 5-point Likert-type scale was used, ranging from “not at all true for me” to “very true for me,” with “neither true nor untrue for me” as the neutral response. When the participants did not understand the item, they could also respond with “I don’t understand this statement.” In Multimedia Appendix 1, an overview of all items can be found with the descriptive statistics. The internal reliability of each skill factor was assessed using Cronbach α: .73 for operational internet skills, .76 for mobile internet skills, and .67 for information internet skills.

After completing the questionnaire, the participants received the activity tracker and downloaded and installed in the corresponding app on their smartphones. The participants used their own smartphone to assure familiarity with the operating system (iOS, Android, or Windows). After installation, the experimenter explained the functions of the activity tracker by showing how to retrieve general data and start tracking sports activities on the device itself. This was followed by an explanation of the app by showing the dashboard: a general overview of the data per topic. The participants were encouraged to go through the dashboard themselves and click on the different data topics (eg, sleep) to check the more detailed data representations (eg, infographics of sleep duration, phases, and schedule). In addition, they were provided with instructions for the first week to get acquainted with the features and data regarding exercise and sleep and to integrate using the activity tracker and its app in their daily lives. Whether the participants followed this instruction was checked by analyzing data on its completeness (eg, the absence of substantive gaps in continuous measurements and regular data synchronization).

#### Wave 2

Wave 2 took place after 1 week of using the activity tracker. This part consisted of 25 tasks regarding data IoT skills, focusing on the retrieval and interpretation of data—gathered by the activity tracker of the participants—using the app. An example of such a data IoT skill task was, “Have you had enough deep sleep if you compare it to other (wo)men your age?” The tasks were distributed across 9 assignments, each covering a different topic (eg, sleep, heart rate, and training). For 2 of the assignments—*Sufficient exercise* and *Good night’s sleep*—the participants also had to compare their data with 9 general health guidelines on exercise and sleep [45,46]. The participants did this by answering questions such as, “Have you been active for at least 150 minutes since wearing the Fitbit?” These questions were answered by filling in yes or no and providing a specific number, in this case, the number of active minutes. Answers to the assignments could be found in the Fitbit app of the participants but were administered on hand-outs to avoid unnecessary switching between the Fitbit app and an administrative app. After finishing the data IoT skill assignments, the assignments *Sufficient exercise* and *Good night’s sleep* were discussed, as the participants could use these assignments for the strategic IoT skill assignment discussed in the following paragraph. An overview of all data IoT skill tasks can be found in Multimedia Appendix 2.

The data IoT skill assignments were followed by a written assignment consisting of 18 tasks measuring strategic IoT skills. This assignment required the construction of a personal action plan based on the (discussed) comparisons made during the data IoT skill assignments *Sufficient exercise* and *Good night’s sleep*. The construction of the action plans for the participants followed the instructions “Use all of the guidelines (9) to find your points of improvement regarding exercise and sleep” and “Explain for each point of improvement how you are planning on improving/executing it” (Multimedia Appendix 2). In other words, 9 tasks involved setting goals for all the guidelines the participants did not yet conform to (eg, not being active for 150 mins a week), and 9 tasks involved describing how they were planning on reaching these goals. After completing the action plan, the answers were discussed with the experimenter and supplemented when incomplete (eg, when a goal or its execution was missing).

All assignments were pilot tested with 6 participants of different ages and educational levels to ensure comprehensibility and applicability. The participants themselves decided when they had finished or wanted to give up on an assignment. However, for the data IoT skill assignments, a time limit was set, after which the participants were asked to pass on to the next assignment. All participants completed the assignments in the same order. The order and the maximum time allowed for the data IoT skill assignments can be found in Multimedia Appendix 2.

#### Wave 3

In wave 3, the execution of the (discussed and adjusted) personal action plan was evaluated. The participants had to evaluate whether they had met their personal goals. The setup of these tasks was similar to that of the data IoT skill tasks, which involved comparing personal data with general guidelines, but instead of comparing the data with the guidelines, the participants compared the data with their personal goals (Multimedia Appendix 2). No time limit was set, as this assignment was person specific.

### Data Analysis

To determine the levels of IoT skills of the participants, we focused on successful task completion of the data IoT skill tasks and of the tasks regarding the strategic IoT skill components, namely, action plan construction and action plan execution. To identify the factors influencing the levels of IoT skills, linear regressions were conducted for data IoT skills and for the 2 strategic IoT skill components, with total task completion scores as the dependent variable. The independent variables in the regression models were gender; educational level attained (coded from low to high); age (years); and the participant’s operational, mobile, and information internet skill levels.

## Results

### Levels of IoT Skills

The levels of IoT skills were determined by successful task completion. As shown in Table 2, on average, the participants completed 54% (13.5/25) of the data IoT skill tasks successfully. Regarding strategic IoT skill tasks, completion rates were slightly higher for action plan construction. On average, 56% (10.1/18) of the construction tasks were completed successfully. For action plan execution, this was 43% (3.9/9).

**Table 2:** Overview of successful task completion.

IoT^a^ skills		Task completion		
		Mean (SD)	Percentage (%)	Minimum to maximum
Data IoT skills		13.5 (4.93)	54	3-22
**Strategic IoT skills**				
	Action plan construction	10.1 (3.96)	56	0-18
	Action plan execution	3.9 (1.46)	43	1-8

^a^IoT: internet-of-things.

None of the participants were able to successfully complete all the data IoT skill tasks. The task that proved to be most difficult was finding the heart rate (bpm) that belonged to the *fat burning* zone threshold, as presented in the activity tracker’s app. Overall, 22.0% (22/100) of the participants were able to complete this task successfully. Regarding strategic IoT skill tasks, 5.0% (5/100) of the participants were able to construct an action plan that included all the guidelines applicable to the participant. They struggled the most with creating a plan to reach their goal regarding active hours—the number of hours they intended to take at least 250 steps. A total of 21.0% (21/100) of the participants recognized how they could reach this goal. In addition, none of the participants were able to execute the constructed action plan. An overview of the number of data and strategic tasks failed can be found in Multimedia Appendix 3.

### IoT Skill Determinants

Table 3 contains the linear regression results of the number of data IoT skill tasks completed successfully (*R^2^*=0.50, *F_6,99_*=15.44; *P*<.001). Age is the strongest contributor, followed by education. This indicates that older people and people with lower levels of education had the most trouble accessing and interpreting data gathered by the activity tracker. In addition to age and educational level, the possession of information internet skills was found to contribute to data IoT skill task completion.

**Table 3:** Data internet-of-things skill task completion.

IoT^a^ skills	Task completion	
	β	*P* value
Gender (male/female)	−.13	.10
Age	−.61	<.001
Education (low/middle/high)	.25	.002
Operational internet skills	−.17	.10
Mobile internet skills	.11	.26
Information internet skills	.17	.03

^a^IoT: internet-of-things.

Table 4 presents the linear regression results of the number of successfully completed strategic IoT skill tasks, both for action plan construction (*R*^2^=0.31, *F_6,99_*=6.95; *P*<.001) and execution (*R^2^*=0.04, *F_6,99_*=.71; *P=*.64). For action plan construction, age and education were significant contributors to task completion. Older and lower-educated people experienced the most difficulty in constructing their own action plans. They experienced the most difficulty with recognizing how they could reach their goal regarding the number of active hours. Unlike data IoT skill task completion, internet skills did not contribute to the successful construction of an action plan. For the strategic IoT skills component of action plan execution, none of the determinants contributed to task completion.

**Table 4:** Strategic internet-of-things skill task completion.

IoT^a^ skills	Task completion			
	Action plan construction		Action plan execution	
	β	*P* value	β	*P* value
Gender (male/female)	−.06	.46	−.004	.97
Age (years)	−.43	<.001	−.11	.29
Education (low/middle/high)	.27	.004	−.02	.84
Operational internet skills	−.07	.58	−.03	.84
Mobile internet skills	−.08	.50	.05	.70
Information internet skills	.16	.09	−.17	.12

^a^IoT: internet-of-things.

### Hypotheses

Table 5 provides an overview of the hypotheses. Hypothesis 1—that there are no differences in data and strategic IoT skill levels between men and women—is supported. Gender did not appear to contribute significantly to any of the IoT skills.

Hypothesis 2—that age contributes negatively to data and strategic IoT skills—is partly supported. The older participants performed poorly compared with the younger participants with regard to data IoT skills and strategic action plan construction. However, age did not appear to be a significant contributor to the level of strategic action plan execution.

Hypothesis 3—that education contributes positively to data and strategic IoT skills—is partly supported. It appears that the level of education affects the data IoT skills and strategic IoT skills regarding action plan construction.

Surprisingly, hypothesis 4a—that operational internet skills contribute positively to data and strategic IoT skills—and hypothesis 4b—that mobile internet skills contribute positively to data and strategic IoT skills—are rejected. It appears that possessing higher levels of operational or mobile internet skills does not contribute to the level of any of the IoT skills. However, hypothesis 4c—that information internet skills contribute positively to data and strategic IoT skills—is supported for data IoT skills. As expected, those in possession of higher levels of information internet skills also possess higher levels of data IoT skills.

**Table 5:** Overview of supported and rejected hypotheses regarding data and strategic internet-of-things skills.

Hypotheses	Validation		
	Data IoT^a^ skills	Strategic IoT skills	
		Action plan construction	Action plan execution
H1^b^: There are no differences of data and strategic IoT skill levels between men and women.	Supported	Supported	Supported
H2: Age contributes negatively to data and strategic IoT skills.	Supported	Supported	Rejected
H3: Education contributes positively to data and strategic IoT skills.	Supported	Supported	Rejected
H4a: Operational internet skills contribute positively to data and strategic IoT skills.	Rejected	Rejected	Rejected
H4b: Mobile internet skills contribute positively to data and strategic IoT skills.	Rejected	Rejected	Rejected
H4c: Information internet skills contribute positively to data and strategic IoT skills.	Supported	Rejected	Rejected

^a^IoT: internet-of-things.

^b^H: hypothesis.

## Discussion

### Principal Findings

In this study, we used activity trackers to examine the levels of IoT skills of Dutch citizens. By using this smart health device, a valid and realistic perspective was provided on how people make sense of IoT data, make informed data-driven decisions, and act accordingly. To do this, people rely on 2 skill sets in particular: data and strategic IoT skills. For health IoT, these skills are necessary to monitor the present health condition and make decisions regarding health maintenance or improvement.

The potential benefits of using health IoT are promising, and they will probably become even more so as the IoT continues to develop. However, to what extent do Dutch citizens possess the skills that are needed to use the IoT beneficially? We addressed this question by measuring actual IoT skills using a performance test, a measure known for its high validity. In addition to the distinction made in this performance test between data and strategic IoT skills, strategic IoT skills were further divided into action plan construction and action plan execution to account for all competences of strategic IoT skills, ranging from setting realistic goals and making data-driven decisions to executing these decisions and reflecting on the progress made toward the goals. Overall, our results suggest that the Dutch population possesses insufficient data and strategic IoT skills. Citizens had significant problems retrieving and interpreting collected data, and they experienced even more difficulty in using the data to make and act upon decisions. However, the successful completion of half of the tasks suggests that the population is, at least to some extent, able to make sense of simple data and make decisions accordingly.

There is a sequential relationship between data and strategic IoT skills, as understanding the collected data are required to make the right decisions. To measure strategic IoT skills independently, in this study, we ensured that the construction of an action plan was discussed afterward and supplemented when incomplete. Only a few succeeded in constructing a complete action plan by themselves. This suggests that the actual number of participants who successfully completed the strategic IoT skills in regard to action plan execution was much lower when no support was provided.

Overall, our results underscore the need for skills development among Dutch citizens regarding the use of health IoT. When citizens possess sufficient IoT skills, certain health issues can be diagnosed and treated prematurely. However, with the present IoT skill levels, Dutch citizens miss out on these opportunities, with all the consequences this entails. In addition to health implications, possessing sufficient IoT skills also has financial implications. For instance, by incorporating insurance companies into the IoT network, citizens can save money on their health insurance [2]. In turn, insurance companies can use IoT-generated data to predict treatment costs across the Dutch population and change charges accordingly. However, at present, IoT data do not provide a fair representation of the Dutch population, as citizens lacking the skills to properly use the IoT are left out of the equation [29].

Both older individuals and lower-educated people appear to possess the least developed data and strategic IoT skill levels. This is problematic, as they could potentially benefit the most from using the health IoT. For instance, for older individuals and lower-educated people, an activity tracker could be a useful tool to track physical activity, as activity levels tend to decline with age [47], and lower-educated people are generally less active during their leisure time [48]. This physical inactivity poses a health risk and makes them prone to chronic diseases. When used correctly, activity trackers can promote healthy exercising behaviors, such as walking, cycling, and running. They can help with self-monitoring activities and general health condition and support goal setting and execution [47]. However, as older and lower-educated citizens lack the skills to use the IoT for these purposes, they miss out on the benefits the activity tracker has to offer.

The role of internet skills regarding the possession of IoT skills appeared to be smaller than expected. Only information internet skills contributed to the possession of IoT skills. These internet skills remain useful, as IoT users still have to retrieve and interpret information to act strategically. Skills such as revisiting a (web) page and understanding a website’s structure remain relevant for using the IoT. Furthermore, information internet skills can directly be used to browse the internet for information regarding where to find data in the IoT system and how to read it. Arguably, the lack of a contribution of operational and mobile internet skills to data and strategic IoT skills can be ascribed to the autonomous character of the IoT, a technology affordance that partly overcomes the lack of required individual skills. Despite the initial setup, fewer operational and mobile skills are needed as no interference by the user is needed to gather the data that can ultimately be used strategically.

### Limitations and Future Research

This study provides an overview of general data and strategic IoT skill levels among Dutch citizens. However, only general levels of these skills were considered. Despite the distinction between action plan construction and execution when testing strategic IoT skills, further research is necessary to identify the participants’ possession of all different facets of data and strategic IoT skills that are needed to handle IoT data and use it strategically.

Furthermore, other skills should be considered in addition to data and strategic IoT skills. Although skills such as operational and communication IoT skills are not apparent during the entire process of using the IoT because of the autonomous character of the IoT, they remain relevant for the setup of IoT devices and actively sharing (autonomously constructed) content, respectively [2]. Moreover, future studies should pay attention to skills related to data privacy, as the IoT network has a significant impact on people’s privacy and potential exploitation by, for example, insurance companies. Using the IoT involves handling an enormous amount of personal information. Without the skills to protect personal data or mitigate potential risks, there is a serious threat from both people with malicious intentions and third parties looking for financial exploitation [2].

For a fair comparison of IoT skills, we began this study with participants with no previous experience with activity trackers. However, 1 week of practice might not have been sufficient for some of the included participants, for example, the older population and lower-educated participants. To counteract such effects, we provided comprehensive user support at the start of the study, including information on all the different functions of the activity tracker. For future research, we recommend using previous experience as a controlling variable.

Furthermore, in the realm of digital inequality, other determinants besides gender, age, and educational attainment should be studied, as previous research regarding digital inequality also found other factors that contribute to differences in skills possession (eg, social or personal factors: [30]). Particularly interesting would be the inclusion of information about the health lifestyles of the participants. Disparities in the ability to use IoT data and to act on propositions made by the IoT are not only a matter of possessing skills but also of health attitudes and behaviors. Similar to skills, health lifestyle depends on social determinants that create differences in the ability to maintain or improve health and to use health services when falling ill [49]. Therefore, we suggest including questions to explore the health attitudes and behaviors of the participants. Furthermore, we recommend performing a longitudinal study to obtain a view of the role of IoT skills in incorporating the IoT in the health lifestyles of participants.

Finally, using an activity tracker as a means to measure general IoT skills should be treated with caution. Despite being one of the most popular IoT applications, they do not embody all the different functions and possibilities that other IoT devices offer. Hence, we cannot draw firm conclusions regarding the generalizability of our findings. In future studies, we recommend studying IoT skills using other health IoT devices or IoT devices from other domains (eg, smart homes). Furthermore, we recommend increasing the number of devices added to the network (eg, a smart scale, blood pressure monitor, and thermometer) when studying IoT skills. This is critical, as adding devices also adds complexity, which, in turn, can increase inequality, as people with lower levels of IoT skills are unable to cope with increasing levels of IoT complexity.

### Conclusions

This study found that the data and strategic IoT skills of Dutch citizens are underdeveloped to benefit optimally from the health IoT and its potential. This is worrisome, as these skills are vital for searching and dealing with the continuous stream of personal health-related data and for making data-based decisions to maintain or improve the present health condition. Performing these actions appears most problematic for the people who could benefit the most from the health IoT: the older and lower-educated populations. These results indicate that policy makers that aim at reducing the digital health divide should aim at improving the level of data and strategic IoT skills, with special attention to older and lower-educated people. Attention for policy should come from both supply (eg, private sector suppliers that develop and use design guidelines for interface designs that are adapted to the abilities of the intended users) and demand (eg, governmental interventions that address educational curricula or forms of public support).

## Appendix

Multimedia Appendix 1 Internet Skills Scale items.

Multimedia Appendix 2 Assignments.

Multimedia Appendix 3 Number of tasks failed per skill set.

## Abbreviations

IoT: internet-of-things
