# Supplementary material for: Internet-of-Things Skills Among the General Population: Task-Based Performance Test Using Activity Trackers
Source: JMIR Hum Factors. 2020 Nov 18;7(4):e22532. doi: 10.2196/22532 (PMC7710448; doi:10.2196/22532)
Supplement: Multimedia Appendix 1 [file humanfactors_v7i4e22532_app1.docx]

**Multimedia Appendix 1**

Internet Skills Scale items

|  | M | SD |
| --- | --- | --- |
| **Operational Internet skills (α=.73)** |  |  |
| I know how to open a new tab in my browser | 4.91 | .45 |
| I know how to use shortcut keys (e.g., CTRL-C for copy, CTRL-S for save) | 4.44 | 1.15 |
| I know how to bookmark a website | 4.43 | 1.23 |
| I know how to download/save a photo I found online | 4.86 | .55 |
| I know how to open downloaded files | 4.90 | .33 |
| **Mobile Internet skills (α=.76)** |  |  |
| I know how to install apps on a mobile device | 4.84 | .63 |
| I know how to compare mobile apps to choose the best option | 4.35 | 1.01 |
| I know how to keep track of the costs of mobile app use | 4.03 | 1.38 |
| I know how to download apps to my mobile device | 4.73 | .86 |
| I know how to upload a photo or video from my mobile device to social media | 4.72 | .88 |
| I know how to switch off the location on my mobile device | 4.72 | .88 |
| **Information Internet skills (α=.67)** |  |  |
| The way in which many websites are designed confusing (R) | 3.14 | 1.29 |
| I find it hard to find a website I visited before (R) | 4.54 | .89 |
| I get tired when looking for information online (R) | 4.36 | 1.09 |
| Sometimes I end up on websites without knowing how I got there (R) | 4.03 | 1.36 |
| I find it hard to decide what the best keywords are to use for online searches (R) | 4.29 | 1.02 |
| *Note: R = reversed.* |  |  |
