# Supplementary material for: Internet-of-Things Skills Among the General Population: Task-Based Performance Test Using Activity Trackers
Source: JMIR Hum Factors. 2020 Nov 18;7(4):e22532. doi: 10.2196/22532 (PMC7710448; doi:10.2196/22532)
Supplement: Multimedia Appendix 2 [file humanfactors_v7i4e22532_app2.docx]

## Multimedia Appendix 2

### Data IoT skills assignments

#### Assignment 1: Get moving (max time allowed: 8 minutes)

1. How many hours did you take at least 250 steps on *[day]* between 9.00-18.00hr?
   Answer: _______ hours
2. What was your longest stationary period on *[day]*?
   Answer: _______ hours_______ minutes

#### Assignment 2: Take a walk (max time allowed: 8 minutes)

1. How many steps have you taken on *[day]* between *[time interval of 15 minutes]*?
   Answer: _______ steps
2. How many meters have you covered on *[same day as previous task]* between *[time interval of 15 minutes]*?
   Answer: _______ meter

#### Assignment 3: Sleep (max time allowed: 8 minutes)

1. How long have you been in deep sleep today?
   Answer: _______ hours_______ minutes
2. Have you had enough deep sleep if you compare it to other *[men/women]* your age?
   Answer:
   □ Yes, because my percentage of deep sleep is in the range of percentages of other *(men/women)* my age
   □ No, because my percentage of deep sleep is below the range of percentages of other *(men/women)* my age
   □ No, because my percentage of deep sleep is above the range of percentages of other *(men/women)* my age

#### Assignment 4: Calories (max time allowed: 8 minutes)

Imagine you want to monitor your weight with the goal not to gain weight. You want to do this by paying attention to the calories you burn. Take into account that *[men/women]* consume *[2.500/2.000]* a day.

1. Have you burned enough calories on *[day]* not to gain weight?
   Answer:
   □ Yes, _______ calories
   □ No, _______ calories

#### Assignment 5: Cardio fitness (max time allowed: 8 minutes)

1. What is your cardio fitness score according to the Fitbit?
   Answer: (between _______ and) _______

Fitbit has divided these scores into the cardio fitness levels: poor, fair, average, good, very good, and excellent.

1. What is your cardio fitness level according to Fitbit?
   Answer: (between _______ and) _______

#### Assignment 6: Heart rate (max time allowed: 8 minutes)

Fitbit uses different heart rate zones to provide insights in your activities. This way, you can train based on these zones. The heart rate zones are fat burn, cardio and peak.

1. From what heart rate starts your heart rate zone ‘fat burn’?
   Answer: From _______ bpm
2. For how long was your heart rate in the heart rate zones ‘fat burn, cardio and peak’ on *[day]*?
   Answer: _______ hours_______ minutes

Besides time in heart rate zones, the Fitbit measures active minutes. The time in heart rate zones is based solely on your heart rate. Active minutes are based on the type of activities and their intensity. It is therefore possible that these amounts of time differ.

1. How many active minutes has Fitbit measured on *[same day as previous task]*?
   Answer: _______ minutes
2. Has Fitbit measured less, as many, or more active minutes compared to the number of minutes in the heart rate zones on *[same day as previous task]*?
   Answer:
   □ Less
   □ as many
   □ more

#### Assignment 7: Training (max time allowed: 8 minutes)

1. Has the Fitbit recorded training activities since you wear it? If so, what kind of training activities did it record?
   Answer:
   □ No
   □ Yes, namely: _______

#### Assignment 8: Sufficient exercise (max time allowed: 15 minutes)

Last week, you have used the Fitbit to monitor your activity. You can compare this information to Fitbit’s health guidelines regarding exercise. These are the exercise guidelines:

- You move/exercise at least twice a week
- You are active for at least 150 minutes a week
- You take at least 10.000 steps a day
- You climb at least 10 floors a day
- You are active at least 9 hours a day (active hour = 250+ steps within an hour)

1. Have you moved/exercised at least twice since wearing the Fitbit?
   Answer:
   □ Yes, _______ times
   □ No, _______ times
2. Have you been active for at least 150 minutes since wearing the Fitbit?
   Answer:
   □ Yes, _______ minutes
   □ No, _______ minutes
3. Have you taken at least 10.000 steps every day since wearing the Fitbit?
   Answer:
   □ Yes
   □ No, I have taken at least 10.000 steps on _______ day(s)
4. Have you climbed at least 10 stairs every day since wearing the Fitbit?
   Answer:
   □ Yes
   □ No, I have climbed at least 10 floors on _______ day(s)
5. Have you been active for 9 hours every day since wearing the Fitbit? (active hour = 250+ steps within an hour)
   Answer:
   □ Yes
   □ No, I have been active for at least 9 hours on _______ day(s)
6. Which of the guidelines have you reached?
   Answer:
   □ You move/exercise at least twice a week
   □ You are active for at least 150 minutes a week
   □ You take at least 10.000 steps a day
   □ You climb at least 10 floors a day
   □ You are active at least 9 hours a day (active hour = 250+ steps within an hour)

#### Assignment 9: Good night’s sleep (max time allowed: 15 minutes)

Last week, you have used the Fitbit to monitor your sleep. You can compare this information to Fitbit’s health guidelines regarding sleep. A number of these guidelines are:

- You fall asleep at a fixed time
- You wake up at a fixed time
- You sleep between 7-8 hours a day
- You get between *[15-25% / 12-23%]* REM sleep

1. Do you have a fixed time you go to sleep?
   Answer:
   □ Yes, around _______:_______. Go to question a
   □ No. Go to question b
   1. How many times did you fall asleep within 30 minutes of this time according to the Fitbit?
      Answer: _______ times
   2. Between what times did you fall asleep according to the Fitbit?
      Answer: Between _______:_______ and _______:_______
2. Do you have a fixed time you wake up?
   Answer:
   □ Yes, around _______:_______. Go to question a
   □ No. Go to question b
   1. How many times did you wake up within 30 minutes of this time according to the Fitbit?
      Answer: _______ times
   2. Between what times did you wake up according to the Fitbit?
      Answer: Between _______:_______ and _______:_______
3. How many days did you manage to get between 7-8 hours of sleep?
   Answer: _______ days
4. Do you, on average, get sufficient REM sleep?
   Answer:
   □ Yes, on average _______%
   □ No, on average _______%
5. Which of the guidelines have you reached?
   Answer:
   □ You fall asleep at a fixed time
   □ You wake up at a fixed time
   □ You sleep between 7-8 hours a day
   □ You get between *[15-25% / 12-23%]* REM sleep

### Strategic skills: action plan construction

#### Assignment (no time limit)

Imagine you want to (ultimately) meet all the health guidelines regarding exercise and sleep. Therefore, you want to construct an action plan to improve your exercise and sleeping habits. Do as follows:

Make an action plan for the following week based on the information you have retrieved with the Fitbit last week.

1. Use all of the guidelines (9) to find your points of improvement regarding exercise and sleep.
2. Explain for each point of improvement how you are planning on improving/executing it

Make sure that your action plan is achievable in one week

### Strategic skills: action plan construction

#### Assignment (no time limit)

Last week, you have made an action plan to improve your exercising and sleeping habits based on general health guidelines:

- You move/exercise at least twice a week
- You are active for at least 150 minutes a week
- You take at least 10.000 steps a day
- You climb at least 10 floors a day
- You are active at least 9 hours a day (active hour = 250+ steps within an hour)
- You fall asleep at a fixed time
- You wake up at a fixed time
- You sleep between 7-8 hours a day
- You get between *[15-25% / 12-23%]* REM sleep

You constructed your own action plan based on these guidelines:

*[Examples]*

- you are active for at least 100 minutes a week
- you sleep at least 7 hours a day
- …

1. Did you manage to be active for at least 100 minutes last week?
   Answer:
   □ Yes, _______ minutes
   □ No, _______ minutes
2. How many days did you manage to get at least 7 hours of sleep last week?
   Answer: _______ days
3. …

The goal of the action plan was to get closer to the exercise and sleep guidelines.

1. Which of the guidelines have you reached last week?
   Answer:
   □ You move/exercise at least twice a week
   □ You are active for at least 150 minutes a week

□ You take at least 10.000 steps a day
□ You climb at least 10 floors a day
□ You are active at least 9 hours a day (active hour = 250+ steps within an hour)
□ You fall asleep at a fixed time
□ You wake up at a fixed time

□ You sleep between 7-8 hours a day
□ You get between *[15-25% / 12-23%]* REM sleep
