# Supplementary material for: Internet-of-Things Skills Among the General Population: Task-Based Performance Test Using Activity Trackers
Source: JMIR Hum Factors. 2020 Nov 18;7(4):e22532. doi: 10.2196/22532 (PMC7710448; doi:10.2196/22532)
Supplement: Multimedia Appendix 3 [file humanfactors_v7i4e22532_app3.docx]

**Multimedia Appendix 3**

Number of tasks failed per skillset

| N of failed tasks | Data skill tasks | Strategic skill tasks |  |
| --- | --- | --- | --- |
|  |  | Action plan construction | Action plan execution |
|  | % of participants | % of participants | % of participants |
| 0 | 0 | 5 | 0 |
| 1 | 0 | 4 | 1 |
| 2 | 0 | 5 | 4 |
| 3 | 1 | 2 | 9 |
| 4 | 5 | 5 | 18 |
| 5 | 5 | 5 | 23 |
| 6 | 8 | 6 | 31 |
| 7 | 4 | 5 | 10 |
| 8 | 9 | 17 | 4 |
| 9 | 6 | 16 | 0 |
| 10 | 12 | 14 | - |
| 11 | 7 | 11 | - |
| 12 | 4 | 7 | - |
| 13 | 6 | 4 | - |
| 14 | 2 | 1 | - |
| 15 | 7 | 0 | - |
| 16 | 5 | 1 | - |
| 17 | 6 | 1 | - |
| 18 | 3 | 1 | - |
| 19 | 1 | - | - |
| 20 | 4 | - | - |
| 21 | 4 | - | - |
| 22 | 1 | - | - |
| 23 | 0 | - | - |
| 24 | 0 | - | - |
| 25 | 0 | - | - |
